# Blindness and Deafness Due to Tape-Worm

**Published:** 1875-10

**Authors:** 


BLINDNESS AND DEAFNESS DUE TO TAPE-WORM.—(*Boston Medical and Surgical Journal, August*, 19, 1875.)—Dr. Williams reports the case of a child of eight years, puny, but in fair health. It had suddenly lost its hearing six weeks previously. Four weeks before, that is, a fortnight after the deafness, it had in one day lost its sight. For a day blindness was complete; then for a time there occurred successive intervals of sight and blindness. If any one whom the child knew brought his eyes close to it, it would catch the expression of the eyes and show recognition, but it recognized no other light, however bright. The ophthalmoscope showed no local trouble or cerebral lesion. There were signs of tape-worm present, and the loss of the two functions could be attributed only to reflex action. There had been no vomiting. On the removal of the worm both sight and hearing returned.—*Medical Times.*

